# High-Dose Oral and Intravenous Rifampicin for the Treatment of Tuberculous Meningitis in Predominantly Human Immunodeficiency Virus (HIV)-Positive Ugandan Adults: A Phase II Open-Label Randomized Controlled Trial

**DOI:** 10.1093/cid/ciab162

**Published:** 2021-03-08

**Authors:** Fiona V Cresswell, David B Meya, Enock Kagimu, Daniel Grint, Lindsey te Brake, John Kasibante, Emily Martyn, Morris Rutakingirwa, Carson M Quinn, Micheal Okirwoth, Lillian Tugume, Kenneth Ssembambulidde, Abdu K Musubire, Ananta S Bangdiwala, Allan Buzibye, Conrad Muzoora, Elin M Svensson, Rob Aarnoutse, David R Boulware, Alison M Elliott

**Affiliations:** 1 Clinical Research Department, London School of Hygiene and Tropical Medicine, Keppel Street, London, United Kingdom; 2 Infectious Diseases Institute, Makerere University, Kampala, Uganda; 3 Medical Research Council - Uganda Virus Research Institute – LSHTM Uganda Research Unit, Entebbe, Uganda; 4 Tropical Epidemiology Group, London School of Hygiene and Tropical Medicine, Keppel Street, London, United Kingdom; 5 Department of Pharmacy, Radboud Institute for Health Sciences, Radboud University Medical Centre, The Netherlands; 6 University of California, San Francisco, San Francisco, California, USA; 7 Division of Biostatistics, University of Minnesota, Minneapolis, Minneapolis, Minnesota, USA; 8 Mbarara University of Science and Technology, Mbarara, Uganda; 9 Department of Pharmacy, Uppsala University, Sweden; 10 Division of Infectious Diseases and International Medicine, University of Minnesota, Minneapolis, Minneapolis, Minnesota, USA

**Keywords:** tuberculous meningitis, rifampicin, intensified therapy, HIV, TBM

## Abstract

**Background:**

High-dose rifampicin may improve outcomes of tuberculous meningitis (TBM). Little safety or pharmacokinetic (PK) data exist on high-dose rifampicin in human immunodeficiency virus (HIV) coinfection, and no cerebrospinal fluid (CSF) PK data exist from Africa. We hypothesized that high-dose rifampicin would increase serum and CSF concentrations without excess toxicity.

**Methods:**

In this phase II open-label trial, Ugandan adults with suspected TBM were randomized to standard-of-care control (PO-10, rifampicin 10 mg/kg/day), intravenous rifampicin (IV-20, 20 mg/kg/day), or high-dose oral rifampicin (PO-35, 35 mg/kg/day). We performed PK sampling on days 2 and 14. The primary outcomes were total exposure (AUC_0–24_), maximum concentration (C_max_), CSF concentration, and grade 3–5 adverse events.

**Results:**

We enrolled 61 adults, 92% were living with HIV, median CD4 count was 50 cells/µL (interquartile range [IQR] 46–56). On day 2, geometric mean plasma AUC_0–24hr_ was 42.9·h mg/L with standard-of-care 10 mg/kg dosing, 249·h mg/L for IV-20 and 327·h mg/L for PO-35 (*P* < .001). In CSF, standard of care achieved undetectable rifampicin concentration in 56% of participants and geometric mean AUC_0–24hr_ 0.27 mg/L, compared with 1.74 mg/L (95% confidence interval [CI] 1.2–2.5) for IV-20 and 2.17 mg/L (1.6–2.9) for PO-35 regimens (*P* < .001). Achieving CSF concentrations above rifampicin minimal inhibitory concentration (MIC) occurred in 11% (2/18) of standard-of-care, 93% (14/15) of IV-20, and 95% (18/19) of PO-35 participants. Higher serum and CSF levels were sustained at day 14. Adverse events did not differ by dose (*P* = .34).

**Conclusions:**

Current international guidelines result in sub-therapeutic CSF rifampicin concentration for 89% of Ugandan TBM patients. High-dose intravenous and oral rifampicin were safe and respectively resulted in exposures ~6- and ~8-fold higher than standard of care, and CSF levels above the MIC.

## Teaser

Rifampicin is undetectable in the cerebrospinal fluid (CSF) of the majority of tuberculosis meningitis patients at standard dosing. Highdose rifampicin administered intravenously 20 mg/kg and orally 35 mg/kg resulted in therapeutic CSF rifampicin concentrations with no excess toxicity in a population consisting predominantly of people living with human immunodeficiency virus (HIV).

Tuberculous meningitis (TBM) is the second leading cause of adult meningitis in sub-Saharan Africa [1, 2], yet to our knowledge no interventional clinical trials have been conducted in African adults with TBM. TBM treatment is extrapolated from pulmonary TB treatment, using rifampicin dosed at 600 mg (8–12 mg/kg/day). The trials conducted in the 1970–80s did not pursue substantially higher doses of rifampicin (Rifampin^®^), largely due to its cost in that era [3]. Rifampicin is highly bactericidal and has important sterilizing activity, and low plasma rifamycin exposures have been linked to poor treatment outcomes and the evolution of resistance [4–9]. A growing body of evidence from animal and clinical studies in pulmonary TB suggests that high-dose rifampicin accelerates bacillary clearance, kills persister organisms, and the maximum tolerated dose in humans is ~40 mg/kg/day [10–14].

Inadequate central nervous system (CNS) drug penetration may be an important contributory factor to TBM mortality. Rifampicin is highly protein-bound, so only ~5% of plasma rifampicin penetrates into cerebrospinal fluid (CSF). With 10 mg/kg/day dosing, the majority of TBM patients have undetectable rifampicin in their CSF [15, 16]. A series of TBM trials have tested higher doses, with a survival benefit observed with intravenous rifampicin at 13 mg/kg/day in Indonesia but not with oral 15 mg/kg/day in Vietnam [15, 17]. As there is first-pass (gut and hepatic) metabolism of oral rifampicin, higher oral dosing of 20 and 30 mg/kg/day has recently been explored in Indonesia. Meta-analysis of the Indonesian data demonstrated a strong relationship between rifampicin total exposure and survival in 148 predominantly HIV-negative TBM patients [18–20]. Yet pharmacokinetics (PK) data from Asia cannot necessarily be extrapolated to African populations due to differences in HIV serostatus, comorbidities, body size, pharmacogenomics, and drug-drug interactions with HIV medicines [21, 22].

We conducted the first randomized clinical trial in African adults with suspected TBM to test the hypothesis that high-dose rifampicin, administered orally or intravenously, is safe and increases blood and CSF exposures and attainment of PK targets in a Ugandan population consisting predominantly of people living with HIV (PLHIV).

## METHODS

### Study Population and Setting

We recruited participants from Kiruddu Hospital in Kampala and Mbarara Regional Referral Hospital in Mbarara, Uganda, as per a published protocol (ISRCTN42218549) [23]. We obtained consent from adults (≥18 years) with suspected TBM and either microbiological confirmation (eg, CSF Xpert MTB/Rif Ultra) or low CSF glucose (CSF:plasma ratio <50% or CSF glucose <65 mg/dL [<3.6 mmol]) with TBM treatment planned. We excluded those with jaundice or known cirrhosis; >3 doses of TB treatment within the previous 3 days; allergy to first-line TB medicines; rifampicin-resistant *M. tuberculosis*; cryptococcosis; relevant drug-drug interaction (eg, HIV protease inhibitors); pregnant or breastfeeding; known porphyria; creatinine clearance <10 mL/min; or unable to attend follow-up visits.

### Randomization

Participants were randomly assigned in a 1:1:1 ratio to 1 of 3 trial arms. The randomization list was generated by a computer-generated permutated block randomization algorithm of different sized blocks using sealed envelopes. Randomization was stratified by site and Medical Research Council (MRC) disease severity grade (grade I or II/III). To avoid delaying urgent TBM treatment, participants were withdrawn and replaced a posteriori if their baseline alanine transaminase (ALT) was >3× upper limit of normal (ULN).

### Study Treatment

Participants were randomized to 1 of 3 antituberculous therapies: (1) IV-20, high-dose intravenous (IV) rifampicin (20 mg/kg/day) administered over 2 hours, alongside oral isoniazid (5 mg/kg), pyrazinamide (25 mg/kg), and ethambutol (20 mg/kg); (2) PO-35, high dose oral rifampicin (35 mg/kg/day), administered as standard fixed-dose combination antituberculous tablets (RHZE) (containing ~10 mg/kg of rifampicin) along with additional 25 mg/kg dose given as 300 mg oral rifampicin capsules given for 8 weeks; (3) standard-of-care (control arm) RHZE tablets (containing ~10 mg/kg/day of rifampicin) according to World Health Organization (WHO) weight-bands (Supplementary materials). In the IV-20 arm, after 14 days, participants were switched to 35 mg/kg oral rifampicin through 8 weeks, as per PO-35 arm. Study drugs were administered under directly observed therapy during hospitalization. Adjunctive corticosteroids were given routinely as dexamethasone 0.4 mg/kg/day IV for week 1, 0.3 mg/kg/day IV for week 2, and thereafter as oral prednisolone 80 mg/day, weaned to a stop over the following 6 weeks [23]. Antiretroviral therapy (ART)-naive individuals initiated ART after completion of the intensive phase of TB treatment (week 8) in accordance with Ugandan guidelines. In Uganda, tenofovir/lamivudine/dolutegravir is the preferred first-line ART regimen since 2018; prior to that efavirenz was the recommended third agent. PLHIV received cotrimoxazole prophylaxis.

### Outcome Assessment and Follow-Up

Participants were reviewed daily during hospitalization for neurological status and adverse events (AE) ascertainment using the Division of AIDS table version 2.1 [24], before being discharged around day 14 unless their medical condition warranted prolonged hospitalization. After hospital discharge, outpatients follow-up occurred at weeks 4, 8, 12, 18, and 24. We assessed neurocognitive performance at weeks 8 and 24, as possible [25, 26]. After 24 weeks, participants were referred to local TB services to complete 9–12 months of therapy.

Primary endpoints were (1) pharmacokinetic parameters in serum (area under the time concentration curve between 0 and 24 hours [AUC_0–24_], maximum concentration [C_max]_) and CSF concentration (C_CSF_); (2) composite safety endpoint during the 8-week intervention period comprising any of: (a) grade 3–5 AEs including drug-induced liver injury; (b) serious AEs (SAEs); or (c) discontinuation of rifampicin for >5 days for any cause. Secondary endpoints included survival to 8 and 24 weeks, time to normalization of consciousness (Glasgow coma scale [GCS = 15]), functional status by modified Rankin scale at 8 and 24 weeks, and quantitative neurocognitive performance Z score at 8 and 24 weeks.

### Pharmacokinetic Analysis

We conducted plasma PK sampling on day 2 (±1) predose and 2, 4, and 8 hours post-dose. We collected a single CSF sample between 2 and 8 hours post-dose with randomized collection windows. On day 14 (±2), we collected a single plasma and CSF sample between 2 and 8 hours post-dose. Total rifampicin concentrations were analyzed by validated high-performance liquid chromatography with ultraviolet detection (HPLC-UV) in the Infectious Disease Institute Translational Laboratory using an LC-2010C HT system (Shimadzu, Kyoto, Japan). The PK parameters C_max_ and area-under-the time-concentration curve up to 8 hours post-dose (AUC_0–8_) were determined using a standard noncompartmental approach with Phoenix WinNonLin (Certara, Princeton, New Jersey, USA) using the log-linear trapezoidal rule. The AUC_0–24_ was determined using a published population PK model modified for IV administration using NONMEM (Icon Development Solutions, Hanover, Maryland, USA) [27]. For further methods see Supplemental materials.

### Statistical Analysis

Sample size was determined following the assumption that PK parameters are normally distributed on the log-scale using rifampicin log-transformed C_max_ standard deviation derived from prior research [15]. PK data from 15 participants per arm achieves 90% power to reject the null hypothesis of equal means. Participants were categorized according to the uniform case definition (definite = microbiologically confirmed, probable = ≥12 points with brain imaging or ≥10 points without brain imaging, possible = 6–11 points with imaging or 6–9 points without imaging, not TBM = <6 points or alternative cause identified) [28]. Statistical analysis adhered to the published protocol [23] and statistical analysis plan. We followed CONSORT guidelines and conducted analyses by intention to treat (ITT). We compared the proportions experiencing grade 3–5 AEs, and the composite safety endpoint during the intervention period, between arms with χ ^2^ test. Mortality at 8 and 24 weeks post-randomization was compared between study arms using the risk difference from a generalized linear regression model with binomial distribution and identity link function. Kaplan-Meier curves were used to compare time to death. Participants withdrawn or lost-to-follow-up were censored at last contact. We compared mean modified Rankin score at 8 and 24 weeks between arms using a linear regression model. Data were collected using DataFax, and analyses were conducted using Stata version 13.1 (StataCorp, College Station, Texas, USA).

### Ethical Considerations and Oversight

Written informed consent was obtained from participants or their caregiver. The trial was approved by the Research Ethics Committees of LSHTM, UK, and Mulago Hospital, Uganda National Council of Science and Technology, and Uganda National Drug Authority. An independent data safety committee reviewed accruing data.

## RESULTS

### Study Population

**Figure 1.**
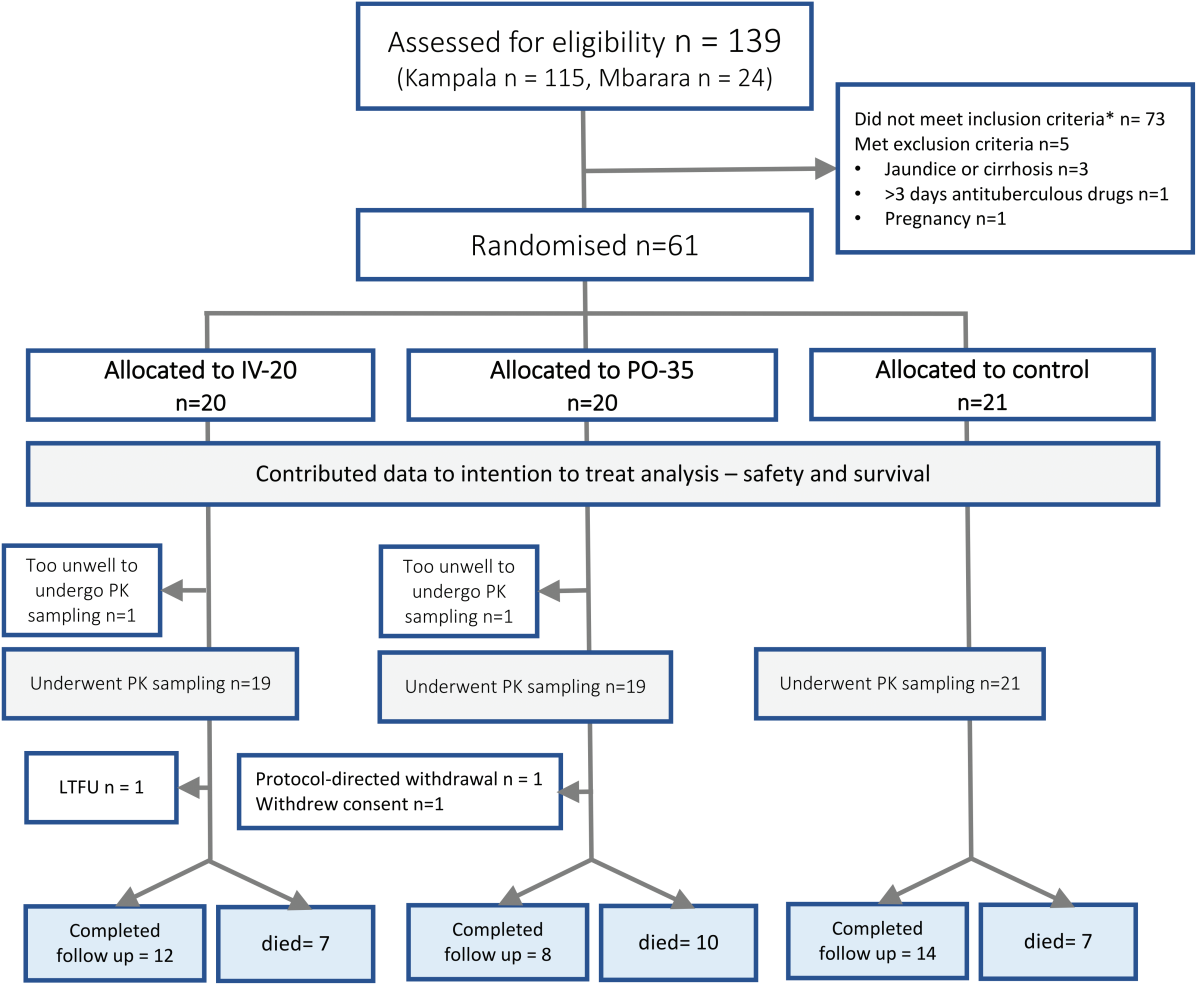
CONSORT diagram. Abbreviations: LTFU, long-term follow-up, PK, pharmacokinetics.

Between 14 January and 17^th^ December 2019, 61 adults with suspected TBM were enrolled, including 31 (51%) with microbiologically confirmed TBM (Figure 1). One participant was withdrawn (from PO-35, baseline ALT > 3× ULN), and an additional participant was enrolled to replace the withdrawal (randomized to the control arm). One participant withdrew consent for follow-up. One participant left hospital against medical advice on day 3 and was lost to follow-up. Baseline characteristics are described in Table 1. The majority of participants (56/61, 92%) were living with HIV with a median CD4 count of 50 cells/µL (interquartile range [IQR] 46–56), a median HIV viral load of 4815 copies/mL, and 20 (33%) were on ART with a median duration of 36 days (IQR 16–61 days). There were no cases of rifampicin-resistant disease identified.

**Table 1.** Baseline Characteristics

	IV-20 Arm	PO-35 Arm	Control Arm
N randomized	20	20	21
Age, median (IQR) years	33.5 (25.5–38.5)	32.5 (26.5–38.5)	34.0 (27– 36)
Gender, male N (%)	13.0 (65)	12.0 (60)	9.00 (42.9)
Weight, median (IQR), kg	55 (47.5–58.5)	50.5 (50–55)	50.0 (45–55)
HIV details			
HIV-positive, N (%)	18.0 (90)	18 (90)	20 (95.2)
HIV-negative, N (%)	2 (10)	2 (10)	1 (4.80)
Amongst those HIV positive:			
CD4 T-cell count, cells/µL	55 (45–59)	50.5 (50–55)	50 (45–55)
HIV viral load, median (IQR) copies/mL	7840 (4014 – 574 462)	6523 (4815 – 92 346)	2334 (945 – 19 316)
Currently receiving ART, N (%)	8 (44)	7 (39)	5 (28)
Antiretroviral therapy duration, median (range), weeks	4 (0 – 62)	3.7 (1.6 – 6.6)	8.7 (0.7 – 196)
MRC TB meningitis grade, n (%)			
I	3 (15)	1 (5)	2 (9.5)
II	13 (65)	16 (80)	12 (57.1)
III	4 (20)	3 (15)	7 (33.3)
Uniform case definition, n (%)			
Definite (microbiologically confirmed)	8 (40)	12 (60)	11 (52.4)
Probable	4 (20)	4 (20)	6 (28.6)
Possible	7 (35)	4 (20)	1 (4.8)
Not	1 (5)	0 (0)	3 (14.3)
CSF information, median (IQR)			
Opening pressure, mmH20	230 (155–320)	220 (135–300)	135 (100–230)
White cells, cells/ µL)	24 (4–162.5)	4 (4–145)	4 (4–122.5)
Protein, mg/dL	154.5 (105–186)	125 (73–188)	92 (30–164)
Glucose, mg/dL	41 (21–68)	44 (21–66.7)	38 (18–64)
Lactate, mmol/L	8.5 (7.30–9.80)	6.9 (2.9–11.1)	8 (5.2–10.4)

Abbreviations: ART, antiretroviral therapy; CSF, cerebrospinal fluid; HIV, human immunodeficiency virus; IQR, interquartile range; MRC, Medical Research Council; TB, tuberculosis.

### Primary Outcomes

**Figure 2.**
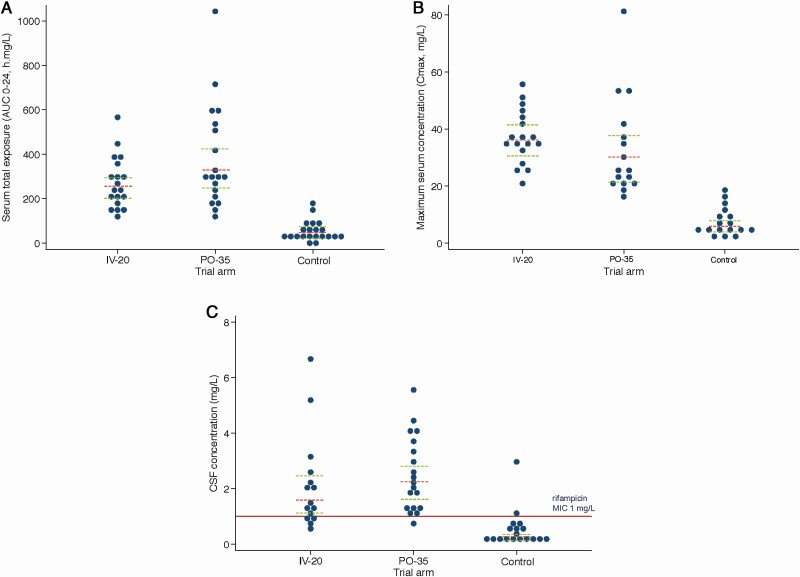
*A-C* Pharmacokinetic parameters on day 2. Distribution of rifampicin. *A*, Area under the concentration-time curve from 0 to 24 hours post-dose (AUC_0–24_), *(B*) maximum concentration in plasma (C_max_), (*C*) CSF concentration (C_CSF_), the horizontal red line is the *Mycobacterium tuberculosis* Uganda II MIC of 1 mg/L. The *x* axis shows the 3 trial arms: intravenous rifampicin 20 mg/kg, oral rifampicin 35 mg/kg, control rifampicin 10 mg/kg, in combination with standard doses of isoniazid, pyrazinamide, ethambutol, and corticosteroids. Red dashed line represents geometric mean concentrations; green dashed lines represent 95% confidence intervals. Abbreviation: CSF, cerebrospinal fluid; MIC, minimal inhibitory concentration.

On day 2 in the standard-of-care arm, serum C_max_ was 6.0 mg/L (95% confidence interval [CI], 4.20–8.68) and AUC_0–24_ was 42.9 h × mg/l (95% CI, 29.2–63.0). With IV-20, C_max_ increased to 36.2 mg/L (95% CI, 31.8–41.2) and AUC_0–24_ increased 5-fold to 217 h × mg/l (95% CI, 202–306; *P* < .001 for each comparison with standard-of-care arm). In the PO-35 arm, C_max_ increased to 29.3 mg/L (95% CI, 23.0–37.5) and AUC_0–24_ increased ~8-fold to 327 h × mg/l (95% CI, 248–430; *P* < .001 for each). In CSF, with standard-of-care treatment 56% (10/18) of participants had undetectable rifampicin (<0.25 mg/L) and geometric mean concentration was 0.27 mg/L (95% CI, <.25–.45). CSF rifampicin was detectable in all participants in the intensified arms. Geometric mean CSF concentrations were ~6-fold higher with IV-20 at 1.74 mg/L (95% CI, 1.20–2.53) and 8-fold higher with PO-35 at 2.17 mg/L (95% CI, 1.64–2.86; *P* < .001 for each). A CSF concentration > 1 mg/L, the rifampicin minimal inhibitory concentration (MIC) for the predominant *M. tuberculosis* strain in Uganda [29], occurred in 11% (2/18) with standard-of-care treatment, 93% (14/15) with IV-20, and 95% (18/19) with PO-35 (*P* < .001). PK results are further described in Table 2 and Figure 2.

**Table 2.** Rifampicin Pharmacokinetic Data by Treatment Arm

	IV-20	PO-35	Control	*P* value^a^
AUC_0–8_ (h × mg/l)^b^				
n observations^c^	19	19	20	
Geometric mean (95% CI)	163 (142–186)	162 (129–203)	30.5 (21.7–42.8)	<.001
Ratio to control	5.33	5.31	…	
*P* value^d^	<.001	<.001	…	
AUC_0_24_ (h × mg/l)^e^				
n observations	19	19	21	
Geometric mean (95% CI)	249 (202–306)	327 (248–430)	42.9 (29.2–63.0)	<.001
Ratio to control	5.80	7.62	…	
*P* value^d^	<.001	<.001	…	
C_max_ (mg/L)^b^				
n observations^g^	19	16	17	
Geometric mean (95% CI)	36.2 (31.8–41.2)	29.3 (23.0–37.5)	6.04 (4.20–8.68)	<.001
Ratio to control	5.99	4.86	…	
*P* value^d^	<.001	<.001	…	
n (%) achieving TDM target of >8 mg/L	20 (100)	20 (100)	10 (47.62)	<.0001^f^
T_max_ (hours)				
Median (range)	2.35 (1.83–3.85)	4.05 (2.17–7.33)	2.83 (2.08– 8.25)	.002^g^
C_CSF_ (mg/L)				
n observations	15	19	18	
Geometric mean (95% CI)	1.74 (1.20–2.53)	2.17 (1.64–2.86)	.27 ^h^ (.17– .45)	.058
Ratio to control	6.44	8.00	…	
*P* value^d^	<.001	<.001		
n (%) with detectable CSF level	15 (100)	19 (100)	8 (44)	<.001 ^f^
n (%) with concentration above rifampicin MIC (1 mg/L)	14 (93.3%)	18 (94.7%)	2 (11.1%)	<.001 ^f^
Median (IQR) hours post-dose	4.70 (3.28–5.92)	4.55 (3.08–6.20)	4.83 (3.78–5.5)	

Abbreviations: AUC, area under the curve; CI, confidence interval; CSF, cerebrospinal fluid; IQR, interquartile range; MIC, minimum inhibitory concentration; PK, pharmacokinetics; TDM, therapeutic drug monitoring.

^a^ Likelihood ratio *P* value from general linear regression.

^b^ Standard 2-step noncompartmental analysis on Phoenix WinNonLin. Where concentration was still increasing at the last sampling point it was not possible to determine C_max_.

^c^ AUC_0–8_ was based on AUC_0-last_ if T_last_ was sufficiently close to 8 hours.

^d^ Pairwise comparison between experimental arm and control arm from general linear regression

^e^ AUC_0–24_ derived using published population PK model in NONMEM [27]

^f^*P* value by χ ^2^ test.

^g^*P* value by Kruskal Wallis test.

^h^ 10 participants had CSF levels below the lower limit of quantification (LLOQ = 0.25mg/l). A value of 50% of the LLOQ was assigned.

In the CSF at day 14, when rifampicin autoinduction is ~90% established [27], 11% (1/9) of participants in the standard-of-care arm had detectable rifampicin (>0.25 mg/L), whereas 88% (7/8) with IV-20 and 89% (8/9) PO-35 arm had detectable CSF levels. Geometric mean CSF concentrations were 0.57 mg/L (95% CI, .30–1.11, *P* = .001) for IV-20 and 0.45 mg/L (95% CI, .21–.96, *P* = .007) for PO-35, compared to 0.15 mg/L (95% CI, .10–.23) for standard-of-care treatment.

During the 8-week intervention period, grade 3–5 AEs occurred in 15 (71%) of standard-of-care treatment, 10 (50%) of IV-20, and 11 (55%) PO-35 participants (*P* = .342). No participants interrupted rifampicin for >5 days. The composite safety endpoint did not differ between arms (*P* = .342), Table 3. The most common grade ≥3 AE, elevated ALT, occurred in 7 (12%) of participants and was attributed to drug-induced liver injury (DILI), which occurred in 4 (19%) participants with standard-of-care treatment, 1 (5%) with IV-20, and 2 (10%) with PO-35. ALT elevations were grade 3, except one episode of grade 4 ALT elevation in the standard-of-care arm. Isolated hyperbilirubinemia, a recognized side-effect of rifampicin, occurred in 3 (5%) participants (grade 4) during the intervention.

**Table 3.** Adverse Events by Treatment Arm During the 8-Week Interventional Period

	IV-20 (n = 20)	PO-35 (n = 20)	Control (n = 21)	*P* value
Total number of events	N (%)	N (%)	N (%)	
Grade 3	6 (30)	6 (30)	12 (57)	.12
Grade 4	3 (15)	4 (20)	4 (19)	.91
Grade 5	5 (25)	7 (35)	5 (24)	.68
Neurological event				
Cerebrovascular accident	2 (10)	1 (5)	1 (4.8)	
Seizures	1 (5)	2 (10)	2 (9.5)	
Headache	0 (0)	0 (0.0)	1 (4.8)	
Hearing loss	0 (0)	1 (5)	0 (0)	
Neuropathy	0 (0)	0 (0)	1 (4.8)	
Altered mental status	2 (10)	1 (5)	2 (9.5)	
Generalised				
Fever	0 (0)	2 (10)	1 (4.8)	
Rash (Kaposi’s sarcoma)	0 (0)	1 (5)	1 (4.8)	
Gastrointestinal and hepatic				
Abdominal pain	0 (0)	1 (5.0)	0 (0)	
Dysphagia	1 (5)	0 (0)	0 (0)	
Elevated alanine transaminase	1 (5)	2 (10)	4 (19)	
Elevated bilirubin	1 (5)	0 (0)	2 (9.5)	
Other				
Anemia	1 (5)	1 (5)	2 (9.5)	
Hypotension	1 (5)	0 (0)	0 (0)	
Thrombosis	0 (0)	0 (0)	1 (4.8)	
Elevated creatinine	1 (5)	1 (5)	1 (4.8)	
Urinary tract obstruction	0 (0)	1 (5)	0 (0)	
Respiratory distress	1 (5)	2 (10)	3 (14)	
Low sodium	1 (5)	1 (5)	0 (0)	
AEs in relation to pre-specified secondary endpoint				
N (%) with a grade 3–5 AE	10 (50)	11 (55)	15 (71)	.343
N (%) with a serious AE	8 (40)	7 (35)	7 (33)	.899
N (%) of patient with discontinuation of rifampicin for >5 days in week 0–8	0	0	0	
Composite of 1 or 2 or 3	10 (50)	11 (55)	15 (71.4)	.343

Values are N (%). *P* values by χ ^2^ test. All serious adverse effects (AEs) were represented within the grade 3–5 AE row and already included in the composite endpoint.

### Secondary Outcomes

**Figure 3.**
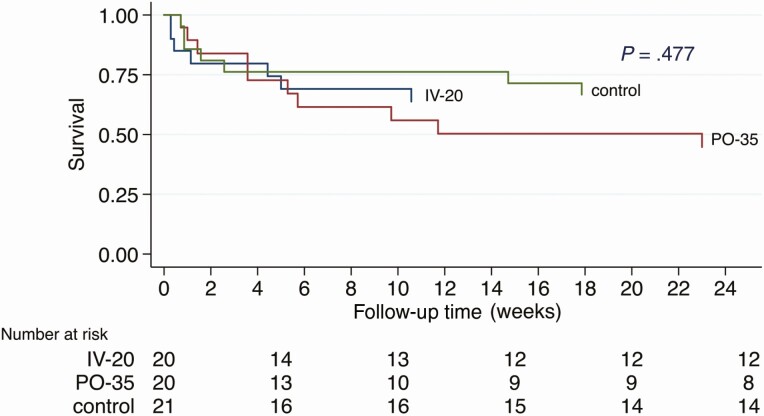
Kaplan-Meier survival. Survival by trial arm. Follow-up time is 24 weeks. Withdrawals (n = 2) and loss to follow-up (n = 1) were censored at their last time of contact. *P* value by log rank test. The study was not powered for survival, and thus mortality was a secondary endpoint.

Overall, during the 8-week intervention period, 18 (30%) participants died: 5/21, 6/20, and 7/20 in the standard-of-care, IV-20, and PO-35 arms, respectively. There was no evidence of an association with treatment arm (LR test *P*-value = .595). By the end of the 24-week follow-up period, 24 (39%) of participants had died: 7/21, 7/20, and 10/20 in the standard-of-care, IV-20, and PO-35 arms, respectively. There was no evidence of association with treatment arm (LR test *P*-value = .333). Kaplan-Meier survival curves are shown in Figure 3. Mean modified Rankin scores (0 = asymptomatic, 6 = dead) at week 8 were 2.06, 1.84, and 2.30 (*P* = .75), and at week 24 were 0.86, 0.81, and 1.14, in the standard-of-care, IV-20, and PO-35 arms, respectively (*P* = .85). Time to normalization of GCS did not differ compared to the standard-of-care arm: IV-20 subdistribution hazard ratio (SHR) 1.20 (95% CI, .58–2.48; *P* = .630) and PO-35 0.85 (95% CI, .41–1.77; *P* = .658). For further results, see Supplementary materials.

## DISCUSSION

In this randomized controlled trial of high-dose rifampicin involving predominantly adults living with HIV with suspected TBM, both high-dose intravenous and oral rifampicin resulted in a far greater proportion of participants achieving CSF levels above the *M. tuberculosis* MIC of rifampicin when compared to standard TB treatment. With the currently WHO-recommended TB therapy, only 1 in 10 people achieved a CSF rifampicin concentration above the MIC. As well as being rapidly bactericidal, rifampicin is the key sterilizing drug in the regimen, so attainment of the rifampicin MIC in the CSF should be considered a bare minimum target in TBM.

Importantly, despite the substantial increase in rifampicin exposure in the intervention arms, there was no increase in toxicity, in line with other studies of largely HIV-negative individuals [10, 15, 18, 30]. The adverse effects of rifampicin are well described and include hypersensitivity reactions featuring fever and/or rash, thrombocytopenia, liver toxicity, and hyperbilirubinemia. Recent data from PanACEA consortium suggest that rifampicin toxicity is largely idiosyncratic, and our findings support the notion that exposure is not a driver of toxicity [14]. This is reassuring given that people with advanced HIV are at increased risk of drug-related toxicity including cutaneous drug reactions, hypersensitivity, liver toxicity, and complications relating to polypharmacy and immune reconstitution [21, 22]. Due to the severely unwell nature of the trial population, grade 3–5 AEs were experienced by over half (59%, 36/61) the participants but were largely complications of the underlying disease process, and events were evenly distributed across arms. Interruption of antituberculous therapy is associated with increased mortality from TBM [31]. We therefore used a DILI management algorithm in which pyrazinamide was interrupted in isolation with regular monitoring of liver function tests, allowing other antituberculous agents to be continued. This was effective in all occurrences of DILI during the intensive phase. We did not observe any impact of high-dose rifampicin on 8- or 24-week mortality, time to normalization of conscious level, nor functional outcomes by modified Rankin scale. This phase II study was not powered for clinical endpoints; thus an adequately powered trial is justified having established the safety of high-dose rifampicin in HIV coinfection.

The rifampicin dosing increases resulted in greater than proportional increases in serum and CSF exposure: a 2-fold dose-increase administered intravenously resulted in ~6-fold increase in exposures, and a 3.5-fold oral dose-increase resulted in a ~8-fold increase in exposures. The supra-proportional increase in exposures observed here is attributable to (1) saturation of the beta-esterase metabolizing enzymes and/or p-glycoprotein with a reduction in the first-pass effect and thereby increased bioavailability of oral drug; (2) saturation of the biliary excretion pathway [10, 27]. Rifampicin induces its own metabolism, a phenomenon known as clearance autoinduction, resulting in lower exposure at steady-state. Despite this phenomenon, we found that rifampicin concentrations in the interventional arms remained higher than those in the standard-of-care arm at day 14 when clearance autoinduction was established.

In relation to other TBM intensification studies, the day 2 total serum rifampicin exposures achieved in the IV-20 and PO-35 arms were, respectively, 3- and 4-fold higher than that of the intensified arm of the landmark Vietnamese trial by Heemskerk et al (AUC_0–24_ 82.5 h × mg/l following rifampicin 15 mg/kg/day) [32]. The total exposure achieved with PO-35 (AUC_0–24_ 327 h × mg/l) is comparable to the most recent Indonesian phase II trial, which tested rifampicin 30 mg/kg/day and reported an AUC_0–24_ of 294 h × mg/l [18]. Owing to the difficulty in consistently culturing *M. tuberculosis*, CSF does not lend itself to microbial kill studies. We therefore cannot draw any parallels with the bacillary clearance rates in pulmonary TB high-dose rifampicin studies, but our data can contribute to a PK-PD model to understand the exposure-response relationship in this population.

Comorbid conditions can impact the PK of antituberculous drugs, including HIV infection, which alters rifampicin exposure in the initial days of therapy. Meta-analysis of rifampicin PK data from 70 studies including 3477 participants confirmed that during the initial days of standard TB treatment rifampicin total plasma exposure is reduced in PLHIV (mean AUC_0–24_ was 37.2 h × mg/l in living with HIV vs 56.7 h × mg/l in HIV-negative adults, *P* = .003), though this difference diminished at steady-state [33]. Our participants in the standard-of-care arm had a serum geometric mean AUC_0–24_ of 42.9 h × mg/l, similar to the PLHIV and lower than the HIV-negative group in the meta-analysis [33]. It is encouraging that despite the study population having advanced HIV, high blood and CSF rifampicin exposures were achieved with oral administration of rifampicin at 35 mg/kg/day. Critical illness can also alter drug PK, compounded by coma requiring TB drugs to be given via nasogastric tube, and vomiting, all of which may jeopardize effective early therapy. It is therefore reassuring that high blood and CSF exposures were achieved with oral rifampicin, which is more widely available in TB endemic settings and a less complex intervention than intravenous rifampicin.

HIV is central to the incidence of TBM in sub-Saharan Africa [34]. The vast majority of the study population (92%) were living with HIV under half were on ART, none were virologically suppressed, and median CD4 count was 50 cells/µL. Effective HIV treatment reduces the risk of TB by ~80%; therefore, earlier and effective HIV treatment are key upstream interventions to reduce the incidence of TBM [35]. Other important upstream interventions include TB preventative therapy and screening for TB prior to ART initiation. The rollout of dolutegravir as first-line ART in Uganda began in 2018 and may support improved HIV virological suppression going forward. We dose-adjusted dolutegravir to 50 mg twice daily, which is known to be effective with standard rifampicin [36, 37]. An ongoing study in Uganda is examining the impact of rifampicin 35 mg/kg on dolutegravir levels [4].

This study has a number of limitations. Due to inability to stand, baseline weight was frequently estimated, potentially introducing imprecision in weight-based dosing. Ideally, we would have collected blood and CSF samples at more time points, especially in the clearance phase, to allow estimation of elimination rate and half-life and CSF C_max_. However, we prioritized participant safety and comfort, so restricted blood sampling to daylight hours and performed a single PK lumbar puncture. As in many TBM trials, microbiological confirmation was only made in half the participants. Because a minority of TBM patients are culture positive, and the complexity of performing rifampicin MIC, the MIC was derived from population estimates within the same TB laboratory [29, 38]. This phase II study was adequately powered for quantification of PK parameters, but due to limited sample size, no conclusions about clinical outcomes can be drawn. Lack of PK-PD target exposures limits the ability to interpret the translational importance of these data.

This phase II study provides encouraging evidence that in a population consisting predominantly of people living with HIV high-dose rifampicin increases CSF and serum exposures with no additional toxicity. These data, taken together with data from Indonesian studies, justify a phase III trial to investigate safety in a wider population and determine the impact of high-dose rifampicin on death and disability. We look forward to the outcome of a number of trials in the pipeline examining high-dose oral rifampicin in isolation (ISRCTN15668391), or with adjunctive linezolid and aspirin (NCT04145258, NCT03927313). Whether in future the optimized TBM treatment regimen will include high-dose rifampicin remains to be concluded.

## Supplementary Data

Supplementary materials are available at *Clinical Infectious Diseases* online. Consisting of data provided by the authors to benefit the reader, the posted materials are not copyedited and are the sole responsibility of the authors, so questions or comments should be addressed to the corresponding author.

ciab162_suppl_Supplementary_Material

## Notes

***Acknowledgments.*** The authors thank the external monitors Ms Mirriam Akello and Ms Sarah Coutinho. Sanofi Pasteur donated the rifampicin to the study. They would like to thank the participants and their caregivers for participating in the study, the Infectious Diseases Institute and LSHTM research office for their support, and the meningitis team for their dedication. F. V. C. is an honorary fellow of the Makerere University—Uganda Virus Research Institute Centre of Excellence for Infection and Immunity Research and Training (MUII-plus). MUII-plus is supported through the DELTAS Africa Initiative (grant number 107743). The DELTAS Africa Initiative is an independent funding scheme of the African Academy of Sciences (AAS), Alliance for Accelerating Excellence in Science in Africa (AESA) and supported by the New Partnership for Africa’s Development Planning and Coordinating Agency (NEPAD Agency) with funding from the Wellcome Trust (grant number 107743) and the UK Government. The MRC/UVRI and LSHTM Uganda Research Unit is jointly funded by the UK Medical Research Council (MRC) and the UK Department for International Development (DFID) under the MRC/DFID Concordat agreement and is also part of the EDCTP2 program supported by the European Union. D. R. B. reports grants from the National Institutes of Health (NIH).

***Disclaimer.*** Sanofi Pasteur donated the intravenous and oral rifampicin. No conflicts of interest were declared by any of the investigators.

***Financial support.*** This work was supported by a Wellcome Clinical PhD Fellowship (210772/Z/18/Z).

***Potential conflicts of interest.*** All authors: No reported conflicts of interest. All authors have submitted the ICMJE Form for Disclosure of Potential Conflicts of Interest.

**Additional Rift study group members.** Members of the trial steering committee: Professor Guy E Thwaites, Professor Reinout van Crevel, Professor Joseph Jarvis.

Members of the data safety committee: Professor Robert J Wilkinson, Dr Christine Sekaggya-Wiltshire, Dr Agnes Kiragga, Dr Amanda Clarke.

Additional contributors to clinical and administrative activities: Jane Frances, Florence Kigundu, Cythia Ahimbisibwe, Carol Karuganda, Alice Namudde, Kiiza Tadeo Kandole, Kathy Huppler Hullsiek, Alisat Sadiq, Mable Kabahubya, Dr Edward Mpoza, Dr Gavin Stead, Dr Samuel Jjunju, Dr Edwin Nuwagira, Dr Nathan Bahr, Dr Joshua Rhein, Darlisha Williams, Rhona Muyise, Eva Laker.
