# Supplementary material for: High-Dose Oral and Intravenous Rifampicin for the Treatment of Tuberculous Meningitis in Predominantly Human Immunodeficiency Virus (HIV)-Positive Ugandan Adults: A Phase II Open-Label Randomized Controlled Trial
Source: Clin Infect Dis. 2021 Mar 8;73(5):876–84. doi: 10.1093/cid/ciab162 (PMC8423465; doi:10.1093/cid/ciab162)
Supplement: ciab162_suppl_Supplementary_Material [file ciab162_suppl_supplementary_material.docx]

**Supplementary material**

# High dose oral and intravenous rifampicin for the treatment

# of tuberculous meningitis in predominantly HIV-positive Ugandan adults: a phase II open-label randomised controlled trial

**Authors:**

Fiona V Cresswell MBChB^1,2,3^, David B Meya PhD^2^, Enock Kagimu MBChB^2^, Daniel Grint PhD^4^, Lindsey te Brake PhD^5^, John Kasibante MBChB^2^, Emily Martyn MBBS^1^, Morris Rutakingirwa MBChB^2^, Carson M Quinn BA^6^, Micheal Okirwoth BSc^2^, Lillian Tugume MBChB^2^, Kenneth Ssembambulidde MBChB^2^, Abdu K Musubire MD^2^, Ananta S Bangdiwala MS^7^, Allan Buzibye MSc^2^, Conrad Muzoora MD^8^, Elin M Svensson PhD^5,9^, Rob Aarnoutse PharmD PhD^5^, Alison M Elliott MD*^1,3^, David R Boulware MD*^10^

**Institutions:**

1. Clinical Research Department, London School of Hygiene and Tropical Medicine, Keppel Street, London, UK
2. Infectious Diseases Institute, Makerere University, Kampala, P.O. Box 22418, Uganda
3. Medical Research Council - Uganda Virus Research Institute – LSHTM Uganda Research Unit, Entebbe, Uganda
4. Tropical Epidemiology Group, London School of Hygiene and Tropical Medicine, Keppel Street, London, UK
5. Department of Pharmacy, Radboud Institute for Health Sciences, Radboud University Medical Centre, Netherlands
6. University of California, San Francisco, California, USA
7. Division of Biostatistics, University of Minnesota, Minneapolis, MN, USA
8. Mbarara University of Science and Technology, Mbarara, Uganda
9. Department of Pharmacy, Uppsala University, Sweden
10. Division of Infectious Diseases and International Medicine, University of Minnesota, Minneapolis, MN, USA

**Corresponding author:**

Dr Fiona Cresswell, Clinical Research Department, London School of Hygiene and Tropical Medicine, Keppel Street, London, WC1E 7HT, UK. Fiona.cresswell@lshtm.ac.uk

**Supplementary methods**

**1. Dosing of rifampicin by trial arm (Table 1a-c).**

R=rifampicin H=isoniazid Z=pyrazinamide E=ethambutol.

**Dosing of isoniazid was ~5 mg/kg/day, pyrazinamide was ~25 mg/kg/day and ethambutol was ~20 mg/kg/day. In the IV-20 group these were given as individual drug formulations. In the other study arm they were part of fixed-dose combination therapy as shown below.**

a) Intravenous rifampicin dosing by body weight

| Weight (Kg) | Rifampicin dose (mg) | SUSPEND RIFAMPICIN IN EACH VIAL (600MG)  IN 10MLS OF WATER FOR INJECTION | Volume used (mL) | Number of vials used | DILUTE IN 500MLS OF 5% DEXTROSE or 0.9% NaCl  ADMINISTER OVER 120 MINUTES |
| --- | --- | --- | --- | --- | --- |
| 28.5 - 31.49 | 600 |  | 10 | 1.0 |  |
| 31.5 – 34.49 | 660 |  | 11 | 1.1 |  |
| 34.5 – 37.49 | 720 |  | 12 | 1.2 |  |
| 37.5 – 40.49 | 780 |  | 13 | 1.3 |  |
| 40.5 – 43.49 | 840 |  | 14 | 1.4 |  |
| 43.5 – 46.49 | 900 |  | 15 | 1.5 |  |
| 46.5 – 49.49 | 960 |  | 16 | 1.6 |  |
| 49.5 – 52.49 | 1020 |  | 17 | 1.7 |  |
| 52.5 – 55.49 | 1080 |  | 18 | 1.8 |  |
| 55.5 – 58.49 | 1140 |  | 19 | 1.9 |  |
| 58.5 – 61.49 | 1200 |  | 20 | 2.0 |  |
| 61.5 – 64.49 | 1260 |  | 21 | 2.1 |  |
| 64.5 – 67.49 | 1320 |  | 22 | 2.2 |  |
| 67.5 – 70.49 | 1380 |  | 23 | 2.3 |  |
| 70.5 – 73.49 | 1440 |  | 24 | 2.4 |  |
| 73.5 – 76.49 | 1500 |  | 25 | 2.5 |  |
| 76.5 – 79.49 | 1560 |  | 26 | 2.6 |  |
| 79.5 – 82.49 | 1620 |  | 27 | 2.7 |  |
| 82.5 – 85.49 | 1680 |  | 28 | 2.8 |  |
| 85.5 – 88.49 | 1740 |  | 29 | 2.9 |  |
| 88.5 – 91.49 | 1800 |  | 30 | 3.0 |  |

b) Rifampicin dosing in high dose oral rifampicin arm

| Weight | Number of RHZE tabs  (150/75/400/275 mg) | Additional R 300mg tablets | Total R dose  (mg) |
| --- | --- | --- | --- |
| 30-37 kg | 2 tabs | 3 tabs | 1200 |
| 38-54 kg | 3 tabs | 4 tabs | 1650 |
| 55-70 kg | 4 tabs | 5 tabs | 2100 |
| ≥ 71 kg | 5 tabs | 6 tabs | 2550 |

c) Rifampicin dosing in the control arm

| Weight | Number of RHZE tablets  (150 / 75 / 400 / 275 mg) |
| --- | --- |
| 30-37 kg | 2 tabs |
| 38-54 kg | 3 tabs |
| 55-70 kg | 4 tabs |
| ≥ 71 kg | 5 tabs |

## 2. Procedures and Laboratory Investigations

CSF was tested at the bedside for Cryptococcal antigen (CrAg LFA, IMMY, Norman, USA), glucose (One Touch Select), and lactate (Nova Biomedical; Waltham, USA). In the local microbiology laboratory, CSF underwent Gram’s stain, bacteriological culture, cell count and differential, protein estimation, and Xpert MTB/Rif Ultra (Cepheid, Sunnyvale, CA). Blood tests were sent to the Infectious Diseases Institute Core Laboratory in Kampala and the Joint Clinical Research Centre Laboratory in Mbarara, Uganda. Baseline bloods included HIV Ab/Ag test, liver function tests, renal function tests, complete blood count, and β-HCG for women. Safety bloods including liver function tests were sent on days 3, 7, 14, 28, and 56, approximately. For pharmacokinetic measurements, we collected blood samples on day two (+/-1) at the following time points: pre-dose and 2-, 4- and 8-hours post-dose. In case of intravenous infusion, the first 2-hour sampling point occurred at the end of the infusion. We collected a single CSF sample on day 2, randomized between 2- to 8-hours post dose with collection windows randomized as 2-4, 4-6, or 6-8-hours post dose. On day 14 (+/-2), we collected a single blood and CSF sample between 2 to 8 hours post-dose for PK analysis. Pharmacokinetic samples were transferred immediately to the Kiruddu Hospital Microbiology Laboratory in Kampala or JCRC laboratory in Mbarara protected from light where they were centrifuged at 3000g for 10 minutes. Following centrifugation, serum or CSF was aliquoted into 1ml cryovials and immediately cryopreserved at -80 ^O^C for later analysis.

## 3. Pharmacokinetic Investigations and analysis

Total rifampicin concentrations were analysed by validated high-performance liquid chromatography with ultraviolet detection (HPLC-UV) in the Infectious Disease Institute Translational Laboratory using an LC-2010C HT system (Shimadzu, Kyoto, Japan). Chromatographic analysis occurred on a Phenyl-Hexyl, 150 × 3 mm ID, 3µm particle size, analytical column (Thermoscientific, USA). The assay was internally validated and performed well in an external international quality control (QC) programme, accuracy of the QC samples was 94 - 102% depending on the concentration. The intra-assay and inter-assay coefficients of variation were 2.9 - 3.6% and 3.4 - 4.8% respectively. The calibration curve for rifampicin in plasma covered a range from 0.25 to 15 mg/L. Samples with concentrations above the upper limit of quantitation were diluted with blank plasma before re-analysis. The accuracy and repeatability of this approach was validated.

The PK parameters C_max_ and area under the time-concentration curve up to 8 hours post dose (AUC_0–8_) were determined using a standard non-compartmental approach with Phoenix WinNonLin (Certara, Princeton, USA) using the log-linear trapezoidal rule. The AUC_0-24_ was determined using a published population PK model [1], modified for IV administration in IV-20 using NONMEM (Icon Development Solutions, Ellicott City, Maryland). T_max_ was defined as the time to reach maximum concentration. The rifampicin CSF concentration (C_CSF_) was the measured concentration in the interval of 2 to 8-hr post dose. Where rifampicin concentrations were below the lower limit of quantification (LLOQ, 0.25 mg/L), for the purposes of PK analysis a value of 0.125 mg/L (half of the LLOQ) was assigned. The difference in log-transformed exposure measures between study arms was assessed in a general linear regression model with the pairwise difference of each arm with the control assessed by the Wald t-test with Bonferroni adjustment for multiple testing. The difference in T_max_ between study arms was tested with Kruskal Wallis test. The between-arm difference in proportion of participants achieving a minimum published C_max_ target of 8 mg/L was compared with a Chi-squared test [2].

**Supplementary results**

**1. Day 14 serum rifampicin concentrations**

Geometric mean exposures had decreased in the arms with oral (but not intravenous) rifampicin, but exposures in the intervention arms remained higher than those in the control arm. The median sample collection time post-dose was 2.67 hours (IQR 2.18–3.17) for serum and 2.83 hours (IQR, 2.18–3.17) for CSF on day 14. The geometric mean concentrations increased from 4.58 mg/L (95%CI, 2.67–7.86 mg/L; n=13) with standard-of-care to 34.2 mg/L (95%CI, 29.2–40.1; n=11; p<0.001) with IV-20 and 15.9 mg/L (95%CI, 6.85–36.9 mg/L; n=13; p=0.003) with PO-35mg/kg.

**2. Individualised AE details (Table 2).**

| **Study arm** | **ID** | **Site** | **AE start date** | **Day** | **Event** | **Diagnosis** | **Grade** | **SAE** | **Relationship to study drug** | **Relationship to HIV** | **Relationship to TBM** | **Outcome of AE** |
| --- | --- | --- | --- | --- | --- | --- | --- | --- | --- | --- | --- | --- |
| IV-20 | 110038 | Kampala | 16/02/2019 | 8 | respiratory distress | aspiration | 3 | no | not related | probably related | probably related | persistent, expected to resolve |
| IV-20 | 110038 | Kampala | 22/04/2019 | 73 | seizures | generalized seizures | 5 | yes | not related | probably related | definitely related | death, unrelated |
| IV-20 | 110038 | Kampala | 25/03/2019 | 45 | seizures | generalized seizures | 3 | yes | not related | definitely related | definitely related | resolved |
| IV-20 | 110038 | Kampala | 16/02/2019 | 8 | seizures | partial seizures | 3 | no | not related | probably related | probably related | persistent, expected to resolve |
| IV-20 | 110065 | Kampala | 13/03/2019 | 1 | stroke | TBM | 5 | yes | not related | definitely related | definitely related | death, unrelated |
| IV-20 | 110102 | Kampala | 15/04/2019 | 0 | altered mental status | TBM | 5 | yes | not related | probably related | definitely related | death, unrelated |
| IV-20 | 110135 | Kampala | 27/05/2019 | 0 | stroke | stroke | 5 | yes | not related | not related | possibly related | severity worsened to grade 5 |
| IV-20 | 110193 | Kampala | 15/09/2019 | 39 | dysphagia | candidiasis | 3 | yes | not related | definitely related | not related | resolved |
| IV-20 | 110220 | Kampala | 30/08/2019 | 1 | altered mental status | TBM | 5 | yes | not related | definitely related | definitely related | severity worsened to grade 5 |
| IV-20 | 110326 | Kampala | 05/12/2019 | 2 | elevated creatinine | acute kidney injury | 3 | no | not related | possibly related | possibly related | resolved |
| IV-20 | 110327 | Kampala | 11/12/2019 | 8 | elevated ALT | DILI | 3 | no | probably related | possibly related | possibly related | resolved |
| IV-20 | 110332 | Kampala | 08/01/2020 | 29 | low sodium | TBM | 4 | yes | not related | not related | probably related | severity worsened to grade 5 |
| IV-20 | 120089 | Mbarara | 11/10/2019 | 9 | low haemoglobin | diarrhoea | 4 | no | not related | probably related | not related | resolved |
| IV-20 | 120089 | Mbarara | 30/10/2019 | 28 | hypotension | sepsis | 5 | yes | possibly related | possibly related | not related | severity worsened to grade 5 |
| IV-20 | 120089 | Kampala | 31/10/2019 | 29 | bilirubin, high | Other | 4 | no | probably related | possibly related | possibly related | chronic, not expected to resolve |
| PO-35 | 110014 | Kampala | 21/01/2019 | 0 | elevated creatinine | acute kidney injury | 4 | no | not related | not related | probably related | resolved |
| PO-35 | 110014 | Kampala | 28/01/2019 | 7 | elevated ALT | DILI | 3 | no | possibly related | possibly related | possibly related | resolved |
| PO-35 | 110071 | Kampala | 25/04/2019 | 35 | abdominal pain | Other | 5 | yes | not related | not related | possibly related | severity worsened to grade 5 |
| PO-35 | 110071 | Kampala | 29/03/2019 | 8 | urinary tract obstruction | Other | 3 | no | not related | not related | not related | persistent, expected to resolve |
| PO-35 | 110124 | Kampala | 24/05/2019 | 11 | hearing loss | hearing loss | 4 | no | not related | possibly related | definitely related | chronic, not expected to resolve |
| PO-35 | 110145 | Kampala | 19/07/2019 | 37 | fever | TBM | 5 | no | not related | probably related | definitely related | severity worsened to grade 5 |
| PO-35 | 110188 | Kampala | 08/01/2020 | 160 | dysphagia | KS disseminated | 5 | yes | not related | not related | definitely related | severity worsened to grade 5 |
| PO-35 | 110214 | Kampala | 03/09/2019 | 12 | low sodium | TBM | 3 | no | not related | probably related | definitely related | chronic, not expected to resolve |
| PO-35 | 110214 | Kampala | 15/09/2019 | 24 | seizures | TBM | 5 | yes | not related | probably related | definitely related | severity worsened to grade 5 |
| PO-35 | 110230 | Kampala | 14/09/2019 | 4 | altered mental status | TBM | 5 | yes | not related | definitely related | definitely related | severity worsened to grade 5 |
| PO-35 | 110269 | Kampala | 20/10/2019 | 4 | stroke | stroke | 4 | yes | not related | probably related | definitely related | chronic, not expected to resolve |
| PO-35 | 110269 | Kampala | 24/10/2019 | 8 | fever | drug fever | 4 | no | possibly related | possibly related | possibly related | resolved |
| PO-35 | 110269 | Kampala | 11/11/2019 | 26 | low haemoglobin | Other | 3 | no | not related | probably related | probably related | persistent, expected to resolve |
| PO-35 | 110291 | Kampala | 07/04/2020 | 154 | vomitting | liver cirrhosis | 4 | yes | not related | not related | not related | chronic, not expected to resolve |
| PO-35 | 110316 | Kampala | 28/11/2019 | 6 | rash | KS disseminated | 5 | yes | not related | definitely related | definitely related | severity worsened to grade 5 |
| PO-35 | 110333 | Kampala | 16/12/2019 | 6 | elevated ALT | DILI | 3 | no | possibly related | possibly related | probably related | resolved |
| PO-35 | 120099 | Mbarara | 10/11/2019 | 6 | respiratory distress | pneumonia, bacterial | 5 | yes | not related | definitely related | definitely related | death, unrelated |
| PO-35 | 120113 | Mbarara | 24/11/2019 | 3 | seizures | partial seizures | 3 | no | not related | possibly related | probably related | resolved |
| PO-35 | 120113 | Mbarara | 29/11/2019 | 8 | respiratory distress | pneumocystis pneumonia | 5 | yes | not related | probably related | probably related | severity worsened to grade 5 |
| Control | 110061 | Kampala | 28/03/2019 | 21 | elevated ALT | DILI | 3 | no | possibly related | not related | not related | resolved |
| Control | 110061 | Kampala | 02/05/2019 | 56 | rash | KS disseminated | 3 | no | not related | definitely related | not related | persistent, expected to resolve |
| Control | 110061 | Kampala | 29/05/2019 | 83 | bilirubin, high | DILI | 4 | no | possibly related | possibly related | possibly related | resolved |
| Control | 110087 | Kampala | 06/04/2019 | 4 | stroke | stroke | 5 | no | not related | probably related | definitely related | severity worsened to grade 5 |
| Control | 110109 | Kampala | 28/04/2019 | 3 | altered mental status | TBM | 5 | yes | not related | probably related | definitely related | severity worsened to grade 5 |
| Control | 110144 | Kampala | 28/06/2019 | 16 | seizures | generalized seizures | 4 | yes | not related | possibly related | probably related | resolved |
| Control | 110144 | Kampala | 24/06/2019 | 12 | neurosensory alteration | TBM | 3 | no | not related | possibly related | not related | persistent, expected to resolve |
| Control | 110144 | Kampala | 01/08/2019 | 50 | respiratory distress | anaemia | 3 | yes | not related | possibly related | probably related | persistent, expected to resolve |
| Control | 110152 | Kampala | 29/06/2019 | 4 | respiratory distress | pneumonia, bacterial | 3 | no | not related | probably related | probably related | resolved |
| Control | 110159 | Kampala | 05/08/2019 | 34 | headache | cryptococcal meningitis | 3 | yes | not related | definitely related | not related | resolved |
| Control | 110177 | Kampala | 10/09/2019 | 50 | elevated ALT | DILI | 3 | no | possibly related | possibly related | possibly related | resolved |
| Control | 110195 | Kampala | 06/10/2019 | 55 | fever | sepsis | 3 | yes | not related | possibly related | not related | resolved with sequelae |
| Control | 110195 | Kampala | 07/10/2019 | 60 | bilirubin, high | Other | 4 | no | probably related | possibly related | possibly related | resolved |
| Control | 110195 | Kampala | 22/11/2019 | 102 | respiratory distress | Other | 5 | yes | not related | probably related | probably related | death, unrelated |
| Control | 110228 | Kampala | 11/09/2019 | 2 | elevated creatinine | acute kidney injury | 3 | no | not related | definitely related | definitely related | chronic, not expected to resolve |
| Control | 110228 | Kampala | 09/09/2019 | 0 | altered mental status | TBM | 5 | yes | not related | probably related | definitely related | severity worsened to grade 5 |
| Control | 110250 | Kampala | 15/10/2019 | 13 | elevated ALT | DILI | 4 | no | possibly related | possibly related | probably related | resolved |
| Control | 110251 | Kampala | 27/11/2019 | 57 | bilirubin, high | Other | 4 | no | probably related | possibly related | possibly related | resolved |
| Control | 110287 | Kampala | 04/12/2019 | 34 | elevated ALT | DILI | 3 | no | possibly related | possibly related | possibly related | resolved |
| Control | 110330 | Kampala | 06/04/2020 | 123 | heart failure | heart failure | 5 | yes | not related | not related | not related | severity worsened to grade 5 |
| Control | 110330 | Kampala | 27/12/2019 | 22 | fever | Other | 3 | yes | not related | probably related | probably related | resolved |
| Control | 110330 | Kampala | 04/02/2020 | 61 | fever | Other | 3 | yes | not related | not related | not related | persistent, expected to resolve |
| Control | 110344 | Kampala | 21/12/2019 | 4 | seizures | TBM | 5 | yes | not related | definitely related | definitely related | severity worsened to grade 5 |
| Control | 120108 | Mbarara | 03/12/2019 | 15 | thrombosis | deep vein thrombosis | 3 | no | not related | possibly related | possibly related | persistent, expected to resolve |
| Control | 120108 | Mbarara | 22/11/2019 | 4 | respiratory distress | aspiration | 5 | no | not related | not related | possibly related | severity worsened to grade 5 |

This table shows all the AEs in the 24-week follow up period. The safety endpoint was based on AEs during the intervention period (week 0-8), these are represented in aggregate in Table 3.

**Figure 1. Adverse event competing risks regressions (supplementary figure 1)**

Time to experiencing an adverse event (AE) was compared between arms using a Fine and Gray competing risks proportional hazards regression model and cumulative incidence functions, with death considered a competing risk. There was no difference in time to experiencing an AE when comparing the intervention arms to the control: IV-20 sub-distribution hazard ratio (SHR) 0.734 (95%CI, 0.33-1.65, p=0.456) and PO-35 1.04 (95%CI, 0.53-2.05, p=0.909).

**Figure 2. Random effects linear mixed model of Glasgow coma scale score by study day.**

In addition, we modelled repeated measures GCS profiles using a linear mixed model with random slope parameter. In the linear mixed model of GCS profiles, GCS increased by 0.13 (95% CI, 0.07–0.18; p=<0.001) per day, with no evidence of a difference by arm (p=0.953).

P=0.953

**References**

1. Svensson RJ, Aarnoutse RE, Diacon AH, et al. A Population Pharmacokinetic Model Incorporating Saturable Pharmacokinetics and Autoinduction for High Rifampicin Doses. Clinical pharmacology and therapeutics **2018**; 103(4): 674-83.

2. Stott KE, Pertinez H, Sturkenboom MGG, et al. Pharmacokinetics of rifampicin in adult TB patients and healthy volunteers: a systematic review and meta-analysis. The Journal of antimicrobial chemotherapy **2018**.

3. US FDA D. Bioanalytical method validation guidance for industry. **2018**.
